# The Effects of Rosiglitazone on Task Specific Anxiety-Like Behavior and Novelty Seeking in a Model of Chronic Adolescent Unpredictable Stress

**DOI:** 10.3389/fnbeh.2022.830310

**Published:** 2022-02-11

**Authors:** Hannah G. Sexton, Nathan A. Olszewski, Mary-Louise Risher

**Affiliations:** ^1^Department of Biomedical Sciences, Joan C. Edwards School of Medicine, Marshall University, Huntington, WV, United States; ^2^Hershel ‘Woody’ Williams Veterans Affairs Medical Center, Huntington, WV, United States

**Keywords:** unpredictable stress, adolescence, rosiglitazone, novelty seeking, anxiety

## Abstract

Adolescence is characterized as a period of increased social behavior, risk taking, and novelty seeking, partly due to ongoing maturation in critical brain areas and the hypothalamic-pituitary-adrenal (HPA) negative-feedback system. During this period there is heightened vulnerability to stress that can drive neuro-immune-endocrine remodeling, resulting in the emergence of maladaptive behaviors that increase susceptibility to alcohol and substance abuse. Here we used a rat model to investigate the impact of chronic adolescent unpredictable stress on a battery of behavioral measures to assess anxiety, novelty seeking, risk taking, depression, and voluntary ethanol consumption while determining whether the PPARγ agonist rosiglitazone can attenuate these effects. Adolescent female rats that experienced stress showed increased risk taking behavior and novelty seeking behavior with no change in ethanol consumption. The administration of rosiglitazone during stress induction attenuated stress-induced cortisol elevation, normalized risk taking behavior in a model anxiety, and attenuated novelty seeking in a task-specific manner. Depressive-like behavior was not impacted by adolescent unpredictable stress or the administration of rosiglitazone. The results from this study demonstrate that exposure to unpredictable stress during adolescence increases the prevalence of maladaptive behaviors that are known to increase susceptibility to alcohol and substance abuse, and that rosiglitazone may be an effective therapeutic to attenuate the emergence of select risk taking and novelty seeking behaviors in females.

## Introduction

Adolescence is characterized as a period of increased social behavior, risk taking, and novelty seeking in both humans and rodents (Spear, 2000). This is in part due to critical brain areas, important for top down inhibitory control, still undergoing development and maturation (Crews et al., 2007), and the hypothalamic-pituitary-adrenal (HPA) negative-feedback system still being established and refined (Engel and Gunnar, 2020). Due to the enhanced plasticity of the brain during this phase of maturation, it has been described as a critical period of vulnerability for addiction and stress (Arnsten and Shansky, 2004; Crews et al., 2007). Developmental exposure to stressful events has been associated with the emergence of psychiatric disorders, such as major depressive disorder, anxiety, PTSD, and bipolar disorder (Alloy et al., 2006; Herbison et al., 2017; LeMoult et al., 2020; al’Absi et al., 2021). The development of stress-induced maladaptive behaviors can also result in the emergence of alcohol and substance abuse, compulsive gambling, and generalized risk taking behavior (Sinha, 2008; Carroll et al., 2017; Teixeira et al., 2017; Duffy et al., 2018; Horak et al., 2021). These data suggest that uncontrolled exposure to stressors during this period of heightened neuronal plasticity may act as an important trigger for neuronal circuit remodeling that persists into adulthood.

Circuit remodeling can occur through complex neuro-immune-endocrine multidirectional interactions that involve stress-induced upregulation of cortisol and the induction of cytokine release through neuroimmune activation (Haddad et al., 2002; Wiranowska and Plaas, 2008). Childhood maltreatment and stress resulting in the increased production of cytokines and other pro-inflammatory markers are known to drive neuronal remodeling that have been associated with numerous psychiatric disorders (Munjiza et al., 2018; Wohleb et al., 2018). Moreover, the stress-induced changes in cytokine regulation can persist into adulthood as demonstrated by Carpenter et al. (2010) in which they show that adults with a history of childhood maltreatment exhibit increased release of IL-6 during acute stress when performing the Trier Social Stress Test (TSST). Cytokine-related neuroinflammation has been demonstrated in animal models of chronic unpredictable stress (López-López et al., 2016) and social defeat paradigms (Esquivel-Rendón et al., 2019), and have been directly linked to the emergence of stress-induced behavioral phenotypes, including depression (Fernandes and Gupta, 2019; Mograbi et al., 2020). Interestingly, rosiglitazone has been shown to have both anti-depressant and anxiolytic-like effects in rodent models (Eissa Ahmed et al., 2009; Guo et al., 2017), suggesting that rosiglitazone has the potential to be an effective therapeutic approach for the prevention of stress-induced behavioral maladaptation.

Interestingly, the potent peroxisome proliferator activated receptor (PPARγ) agonist, rosiglitazone was originally used due to its efficacy in the peripheral nervous system (PNS) for the treatment of type 2 diabetes (Richter et al., 2007). This was primarily due to its ability to increase the insulin sensitivity of skeletal muscle, liver, and adipose tissue without directly stimulating insulin secretion from pancreatic ß-cells, thus reducing plasma glucose levels (Wagstaff and Goa, 2002; Richter et al., 2007). It was also viewed as beneficial due to its efficacy in modulating cholesterol homeostasis and inflammation (Chinetti et al., 2000, 2003; Standiford et al., 2005). More recently, it has been shown that some of the PNS actions of rosiglitazone are mediated through the suppression of NF-κB and through a macrophage shift from an inflammatory phenotype to an anti-inflammatory phenotype (Bouhlel et al., 2007), similar to the process that occurs in microglia within the central nervous system (CNS) (Subramaniam and Federoff, 2017; Ji et al., 2018). These data suggest that the effects of rosiglitazone are likely mediated through multiple complex mechanisms that involve peripheral and central immune cells, endothelial cells, neurons, and glia (Cullingford et al., 1998; Moreno et al., 2004; Bernardo and Minghetti, 2008). The role of neuroinflammation in animal models of chronic unpredictable stress and the direct link to the emergence of multiple stress-induced behavioral phenotypes suggests that rosiglitazone may be the ideal therapeutic intervention to attenuate stress (immune)-induced neuronal remodeling and the emergence of maladaptive, stress-related, behavioral phenotypes that are associated with increased risk of developing alcohol and substance abuse disorder.

In the present study, we investigate the effectiveness of rosiglitazone in preventing the emergence of stress-induced behavioral changes following adolescent chronic unpredictable stress. We hypothesize that the administration of rosiglitazone will attenuate the emergence of stress-induced risk taking and novelty seeking behavior. By administering rosiglitazone during adolescent chronic unpredictable stress, we reveal task-specific attenuation of stress-induced behaviors following a battery of tests to assess anxiety, novelty seeking, depression, ethanol consumption, and risk taking behavior that may contribute to increased vulnerability to substance use disorder and the emergence of other maladaptive behaviors.

## Materials and Methods

### Animals

Young female Sprague-Dawley rats (Hilltop, Scottdale, PA, United States), PND 21, were housed 4 per cage and maintained on a 12/12-h reverse light cycle (lights on at 6:00 p.m.) with access to food and water *ad libitum*. Animals were allowed to acclimatize to the facility for a minimum of 2 days before any experimental procedure was conducted. A total of 32 animals were used for these experiments (8 per treatment group). All procedures were conducted according to NIH guidelines and were reviewed and approved by the Marshall University Institutional Animal Care and Use Committee.

### Drugs

Animals were separated into 1 of 4 groups: no stress + vehicle (No stress + VEH); no stress + rosiglitazone (No stress + ROSI); stress + vehicle (Stress + VEH); stress + rosiglitazone (Stress + ROSI). Beginning on PND 26, animals received 10 mg/kg ROSI (R0106, TCI Chemicals, Portland, OR, United States) or vehicle (2% methylcellulose, M0292, TCI Chemicals, Portland, OR, United States) dissolved in water and administered *via* gavage immediately prior to introduction to each stressor. Administration of ROSI or vehicle was daily and ended on PND 43.

### Behavioral Approaches

#### Pre-stress Behavioral Assessment

Based on performance in the light-dark box and sucrose consumption, animals were pseudobalanced into one of the four treatment groups (as described below) with the goal of being able to ensure that there were no baseline differences between groups.

#### Sucrose Test

On PND 24 animals were individually housed and allowed access to two bottles, one containing tap water and the other containing 1% sucrose, as previously described in Sequeira-Cordero et al. (2019). During this test, animals were individually housed with access to the two bottles and *ad libitum* access to food for 24 h, after which time they were returned to their home cages. Sucrose and water consumption were measured. Bottles containing water and sucrose were also placed in an empty cage to adjust for normal fluid leakage. Sucrose and water consumption was calculated as percentages [(intake ml/200 ml) × 100] and then corrected by body weight (consumption%/body weight in grams). Similarly, preference was calculated as percentages {[sucrose consumption/(sucrose + water consumption)] × 100} and corrected by body weight. The sucrose consumption test was conducted two more times at PND 51 and PND 149 using the same parameters.

#### Light Dark Box

On PND 23 animals were habituated to the testing room for 30 min. Animals were placed in the bright chamber of the light dark box (50 lux on the bright side) and allowed to explore both the light and dark chambers for 10 min (box diameters: 50 cm × 50 cm). Locomotor activity was recorded using Any-maze Video Tracking System Version 7.08 (64-bit, Stoelting, Wood Dale, IL, United States). Following the 10 min. session animals were returned to the home cage. Animals were assessed for time spent in each of the two zones. Entry into a zone was counted when the animal was half-way into the designated zone, i.e., the automated software measured from the center of the body mass.

#### Intermittent Stress Paradigm

On PND 25, based on the pre-stress measures animals were divided into four equal groups (No stress + VEH; No stress + ROSI; Stress + VEH; Stress + ROSI) and either remained in group housing (no stress groups) or were separated into single housing cages (stress groups) of the same dimensions. Beginning on PND 26 and ending on PND 44, animals underwent an intermittent stress paradigm based on Sequeira-Cordero et al. (2019) with modifications. At PND 26 stress animals underwent restraint stress in a plastic restraint tube for 10–15 min and were then returned to the home cage. On PND 27 stress animals were placed in a new cage with wet bedding (1L of water was added to the cage bedding), they remained in this environment for 24hr. Upon completion, animals were returned to their home cages which were immediately placed in the opposite light cycle. 24 h after sleep disruption, animals were returned to the reverse light cycle and were food deprived for 24 h. Following food deprivation, animal cages were tilted at a 45 degree angle for 24 h. Following cage tilting, animals underwent 24 h water deprivation. These stressors were then repeated in a new randomized order in order to complete three cycles of each stressor. Complete order of stressors and restraint times are detailed in Table 1.

**TABLE 1:** Stress paradigm: The stress protocol consisted of the following stressors: restraint stress (15 min); wet bedding: 1000 mL of water was mixed with 1 L of bedding; sleep deprivation: rats were placed in the opposite light cycle but were not prevented from sleeping; food deprivation; cage tilting: the cage was tilted to 45 degrees; water deprivation; and isolation: rats were individually housed in cages (20 cm length × 10 cm width × 13 cm height).

Stress	Time	Age (PND)	Stress treatment
Restraint stress	15 min	26	Restraint then back to home cage
Wet bedding	24 h	27	1L of Water in cage bedding
Sleep deprivation	24 h	28	Placed in light cycle
Food deprivation	24 h	29	No food
Cage tilting	24 h	30	Tilted cage at 45 degree angle
Water deprivation	24 h	31	No Water
Sleep deprivation	24 h	32	Placed in light cycle
Food deprivation	24 h	33	No food
Restraint stress	13 min	34	Restraint then back to home cage
Wet bedding	24 h	35	1 L of Water in cage bedding
Water deprivation	24 h	36	No water
Cage tilting	24 h	37	Tilted cage at 45 degree angle
Water deprivation	24 h	38	No water
Sleep deprivation	24 h	39	Placed in light cycle
Food deprivation	24 h	40	No food
Restraint stress	10 min	41	Restraint then back to home cage
Sleep deprivation	24 h	42	1 L of Water in cage bedding
Cage tilting	24 h	43	Tilted cage at 45 degree angle
Blood draw		44	

*Rats remained in social isolation between stressors and throughout all behavioral testing, unless stated otherwise.*

*The control (CON) groups were group-housed (3–4 rats per cage) throughout the entire experiment, unless stated otherwise.*

*Stressors followed a semi-random order to reduce their predictability.*

*All stressors lasted 24 h, except for restraint sessions.*

*All animals were exposed to every stressor three times throughout the stress paradigm.*

#### Cortisol Assessment

Immediately following the final 24 h stressor (cage tilting), animals had blood drawn *via* the saphenous vein. Blood was placed in Microvette CB 300 EDTA tubes (Fisher Scientific, Hampton, MA, United States) and centrifuged at 8000 rpm for 5 min. Samples were flash frozen in liquid nitrogen and kept frozen until corticosterone levels were quantified using Enzo Life Sciences kit (Catalog # ADI-900-097, NY, United States).

#### Post-stress Behavioral Assessment

##### Elevated Plus Maze

Elevated Plus Maze (EPM) was performed as previously described in Sequeira-Cordero et al. (2019). On PND 45, animals were habituated to the testing room for 30 min (10 lux). Animals were then placed in the center zone of the elevated plus maze (Stoelting, Wood Dale, IL, United States) located 50.8 cm above the floor. Animals were allowed to explore the maze for 5 min. Following completion of the 5 min exploration time, animals were returned to their home cages. Time spent and distance traveled in closed and open arms were recorded using Microsoft LifeCam camera (Microscoft, Redmond, WA, United States). Data were analyzed using Any-maze Video Tracking System Version 7.08 (64-bit, Any-maze, Wood Dale, IL, United States). Animals were placed in the center of the apparatus facing the open arm. An entry was counted when the animal was half way into the designated zone, i.e., the automated software measures from the center of the body mass.

##### Open Field

Open field was performed as previously described in Sequeira-Cordero et al. (2019). On PND 46, animals were habituated to the testing room for 30 min (10 lux). Animals were then placed in center of the open field (dimensions: 40 cm × 40 cm) for 10 min to explore. Upon completion, animals were returned to their home cages. Locomotor activity was recorded using Any-maze Video Tracking System Version 7.08 (Any-maze, Wood Dale, IL, United States). The thigmotaxis zone was defined as the outer 10 cm of the apparatus, around the edge of the testing box.

##### Force Swim

Forced swim was conducted as previously described in Sequeira-Cordero et al. (2019). On PND 47–48, animals were habituated to the testing room for 30 min (30 lux). Animals were then placed in the forced swim apparatus [dimensions: 18 cm (W) × 60 cm (H)] for 15 min on day one for the pre-test and 5 min on day two for the test day. Activity was recorded and analyzed using Any-maze Video Tracking System Version 7.08 (Any-maze, Wood Dale, IL, United States).

##### Novel Object Recognition

On PND 49–51 and PND 157–159 animals were tested on the Novel Object Recognition (NOR) [dimensions: 30 cm (W) × 70 cm (L) × 60 cm (H)], as previously reported in Swartzwelder and Risher (2012) and Sequeira-Cordero et al. (2019). Animals were habituated to the room for 30 min (30 lux) on day 1. On day 2, animals were placed in the NOR with two identical objects [dimensions: 5 cm (W) × 13 cm (H)] and allowed to explore the arena and objects for 5 min. Animals were returned to their home cages after every trial. 24 h later (day 3), animals were placed back in the NOR apparatus for 5 min to explore one familiar object (from the previous day) and one unfamiliar object [novel object, dimensions: 6 cm (W) × 15 cm (H)]. Objects and object location were counterbalanced across groups. Object exploration was recorded using Any-maze Video Tracking System Version 7.08 (Any-maze, Wood Dale, IL, United States) and hand scored by a blinded experimenter using Any-maze. Exploration was defined as whenever the rat’s nose touched the object or when it was directed toward the object within a distance of 2 cm. Animals that managed to move the objects or didn’t explore one of the two objects during day 2 were excluded from the final analysis (two animals were excluded in total). The Discrimination Index was also calculated as [(novel object exploration time − familiar object exploration time)/(novel object exploration time + familiar object exploration time)]. Video files from PND 49–51 were corrupted and therefore could not be used for the analysis.

##### Two-Bottle Choice

Beginning on PND 52, animals began the two-bottle choice paradigm based on Simms et al. (2008) and Fu et al. (2015). Briefly, ethanol (EtOH) solutions were prepared in tap water from 95% (v/v) EtOH (VWR, Radnor, PA, United States). Animals were individually housed and given 24 h access to two bottles, one water and one containing 20% unsweetened EtOH. After 24 h, the ethanol bottle was replaced with a second water bottle that was available for the next 24 h. Upon placement and removal, the bottles were weighed (to the nearest gram). Animals had access to EtOH beginning on Monday, Wednesday, and Friday morning during the dark cycle for a total of 40 sessions. The placement of the EtOH bottle was randomized to prevent place bias.

##### Social Interaction Protocol

The social interaction task was performed as previously described in Kaidanovich-Beilin et al. (2011). Social interaction was performed using a three-chamber social interaction chamber (Stoelting, Wood Dale, IL, United States) that consisted of three 20 (W) × 40 (L) × 22 cm (H) chambers. The stranger rat that occupied the stranger enclosure was habituated to the social interaction apparatus and stranger enclosure for 10 min per day for 5 days (7 cm circular enclosure) in order to limit stress-induced negative vocalizations. Animals to be tested for social interaction were habituated to the testing room for 30 min prior to each session. On day 1, animals were allowed to freely explore the apparatus for 10 min and then returned to their home cage. On day 2, animals were placed in the apparatus and allowed to explore all chambers and the empty stranger enclosures for 10 min. Animals were then returned to their home cages. On day 3, a stranger animal was placed in the stranger enclosure in the unbiased side and test animals were allowed to explore all chambers for 10 min. After 10 min, a new stranger animal was placed in the opposite chamber in a secondary stranger enclosure. Test animals were allowed to explore for another 10 min. Social recognition was measured as the time the test rat spent sniffing the rats within the enclosures over the 10 min period. Animal activity was recorded and analyzed using Any-maze Video Tracking System Version 7.08 (Any-maze, Wood Dale, IL, United States).

### Statistical Procedures

Statistical significance was defined as an α of *p* < 0.05. All statistical analyses were performed using GraphPad Prism 9.0 (GraphPad, San Diego, VA, United States). For all comparisons (unless otherwise stated) a One-way ANOVA was performed followed by *post hoc* Tukey’s multiple comparisons test. For EtOH consumption studies a repeated measures two-way ANOVA was conducted followed by *post hoc* Tukey’s multiple comparisons test.

## Results

### Pre-test Measures (Sucrose Consumption and Light-Dark Box)

**FIGURE 1:**
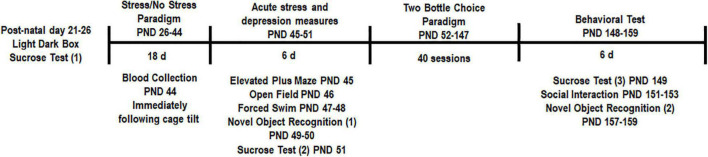
Experimental design: All animals underwent pre-testing in the sucrose consumption test and the light-dark box. Based on these outcomes animals were pseudobalanced into their respective groups (CON + VEH, CON + ROSI, STRESS + VEH, STRESS + ROSI). The chronic unpredictable stress protocol began on PND 26 and ended on PND 43 so as to only encompass adolescence. Body weight was monitored throughout the study. Blood was collected immediately following the final stress procedure (the 24 h cage tilt) to assess cortisol levels. Animals were then tested in the elevated plus maze (PND 46), open field (PND 46), forced swim (PND 47–48), novel object recognition (PND 49–50 and PND 157–159), and a second sucrose consumption test (PND 52). Animals were then tested in the two-bottle choice ethanol paradigm to assess changes in ethanol consumption across 40 sessions. A third sucrose test was conducted (PND 149) followed by the social interaction test (PND 151–153) and the novel object recognition task (PND 157–159).

**FIGURE 2:**
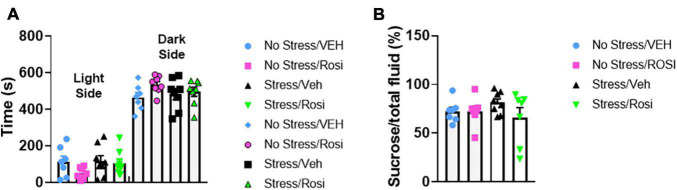
Pre-stress pseudobalancing: At postnatal day (PND) 21–26 rats were screened behaviorally using the sucrose preference test **(A)** and the light dark box **(B)**. Based on the time spent in the open chamber and sucrose preference, the animals were balanced and semi-randomly allocated to the No stress + Vehicle (No stress + VEH), No stress + rosiglitazone (No stress + ROSI), chronic unpredictable stress + Vehicle (Stress + VEH), or the chronic unpredictable stress + rosiglitazone (Stress + ROSI) group. No significant differences between treatment groups were detected in the pre-stress phase, light-dark box: *F*(3,25) = 1.556, *p* = 0.2249 and % sucrose consumption: *F*(3,25) = 1.029, *p* = 0.3966. *n* = 8 per treatment group.

The overall experimental design is shown in Figure 1. Pre-testing was performed to obtain a quick but robust assessment of stress indicators prior to placing animals into stress and treatment groups. Based on statistical analysis we pseudobalanced all groups based on sucrose consumption and anxiety level in the light-dark box. After separation into stress and treatment groups, we were unable to detect any significant differences between groups [time spent in dark chamber, ANOVA, *F*(3,25) = 1.556, *p* = 0.2249, Figure 2A and sucrose consumption, ANOVA, *F*(3,25) = 1.029, *p* = 0.3966, Figure 2B]. One animal from the No stress + ROSI group and one from the Stress + ROSI group died from an unknown cause during the stress paradigm. Three outlier animals were excluded from further testing due to anxiety measures, bringing *n* for the treatment groups to: No stress + VEH = 7; No stress + ROSI = 6; Stress + VEH = 8; and No stress + ROSI = 6.

### Corticosterone Levels

**FIGURE 3:**
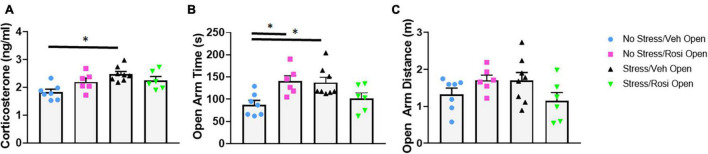
Attenuation of stress induced cortisol upregulation and increased open arm time in the EPM: At PND 44, immediately following the final stress procedure cortisol levels were assessed **(A)** followed by behavioral assessment in the EPM (PND 45, **B**). ROSI significantly attenuated the stress-induced increase in cortisol levels and increased time spent in the open arms of the EPM. There was no effect of stress or treatment on distance traveled in the open arms (**C**, *p* > 0.05). *n* = 6–8 per treatment group. Figures show mean ± SEM. ^∗^ = *p* < 0.05.

Immediately following chronic intermittent stress, animals underwent assessment of blood corticosterone levels to assess whether this stress paradigm was able to induce a physiological stress response. We determined that unpredictable stress (Stress + VEH) resulted in a significant increase in corticosterone levels when compared to controls [ANOVA, *F*(3,23) = 5.979, *p* = 0.0036; Tukey’s *post hoc* analysis, Stress + VEH vs. No stress + VEH, *p* = 0.0018] confirming a physiological stress response in the stress-only animals. Co-administration of rosiglitazone (during stress induction) attenuated the effect of stress on cortisosterone levels (Tukey’s *post hoc* analysis, No stress + VEH vs. Stress + ROSI, *p* = 0.0757, however, Stress + VEH vs. Stress + ROSI, *p* = 0.5242, Figure 3A).

### Anxiety-Like Behavior and Locomotor Hyperactivity: Elevated Plus Maze and Open Field

To determine whether chronic intermittent stress increases anxiety, we assessed performance on the elevated plus maze 1 day after the termination of the stress induction paradigm. Animals that underwent the stress paradigm (Stress + VEH) spent significantly more time in the open arm when compared to controls [ANOVA, *F*(3,23) = 5.187, *p* = 0.007; No stress + ROSI, Tukey’s *post hoc* analysis, *p* = 0.0185, Figure 3B]. Heightened activity in the open arms was attenuated by co-administration of rosiglitazone during the stress induction paradigm (Tukey’s *post hoc*, No stress + VEH vs. Stress + ROSI, *p* = 0.8200, however, Stress + VEH vs. Stress + ROSI, *p* = 0.1563 Figure 3B). While distance traveled in the open arms demonstrated the same trend, i.e., an increase in distance traveled by Stress + VEH animals, however, there was no overall effect [ANOVA, *F*(3,23) = 2.016, *p* = 0.1397, Figure 3C].

**FIGURE 4:**
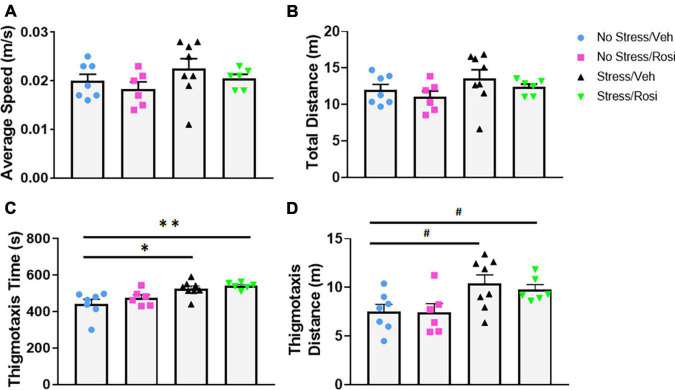
The effects of stress and ROSI on locomotor activity: On PND46 animals were assessed in the open field. There was no overall effect of stress or treatment on overall speed or distance traveled (**A,B**, respectively, *p* > 0.05). Animals exposed to stress spent more time in the thigmotaxis zone (**C**; ANOVA, Tukey’s *post hoc p* = 0.0119) and demonstrated a trend toward increased distance traveled in the thigmotaxis zone (**D**; ANOVA, Tukey’s *post hoc p* = 0.0658); none of these effects were attenuated by ROSI administration (*p* > 0.05). *n* = 6–8 per treatment group. Figures show mean ± SEM. ^∗∗^ = *p* < 0.001, ^∗^ = *p* < 0.05, ^#^ = *p* < 0.07.

As a secondary measure of anxiety and stress-induced locomotor activity, we assessed activity in the open field. There were no overall stress- or treatment-dependent changes in average speed [ANOVA, *F*(3,23) = 1.19, *p* = 0.3355, Figure 4A] or total distance traveled [ANOVA, *F*(3,23) = 1.3, *p* = 0.2983, Figure 4B]. Animals that underwent the stress paradigm (Stress + VEH) spent significantly more time in the thigmotaxis zone when compared to No stress + VEH controls [ANOVA, *F*(3,23) = 6.293, *p* = 0.0028, Tukey’s *post hoc*, *p* = 0.0119, Figure 4C] that was not attenuated by ROSI treatment (Tukey’s *post hoc*, No stress + VEH vs. Stress + ROSI, *p* = 0.0051, Stress + VEH vs. Stress + ROSI, *p* = 0.9268, Figure 4C). This was accompanied by an overall effect on distance traveled in the thigmotaxis zone [ANOVA, *F*(3,23) = 3.665, *p* = 0.0271, Figure 4D]. However, *post hoc* analysis demonstrated only a trend toward an increase in distance traveled when comparing No Stress + VEH vs. Stress + VEH (Tukey’s *post hoc*, *p* = 0.0658, Figure 4D) and No Stress + VEH vs. Stress + ROSI (Tukey’s *post hoc*, *p* = 0.0704, Figure 4D).

### Depression-Like Behavior: Forced Swim and Sucrose Consumption

**FIGURE 5:**
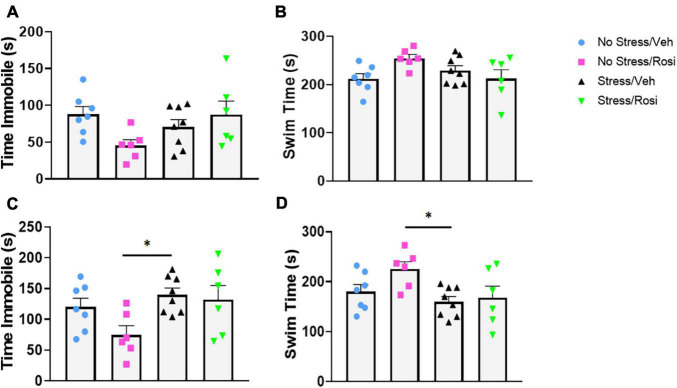
The effects of stress on depressive-like behavior: At PND 47–48 animals were assessed for depressive-like behavior in the forced swim test. There was no significant effect of stress or treatment on immobility or swimming in the pre-test phase **(A,B)**. In the post-test phase there was a significant decrease in immobility in the No Stress + ROSI group when compared to the Stress + VEH group (ANOVA, Tukey’s *post hoc p* = 0.0314, **C**). This was recapitulated in the swim time with No Stress + ROSI treated animals spending more time swimming than the Stress/VEH group (ANOVA, Tukey’s *post hoc p* = 0.0314, **D**). *n* = 6–8 per treatment group. Figures show mean ± SEM. ^∗^ = *p* < 0.05.

**FIGURE 6:**
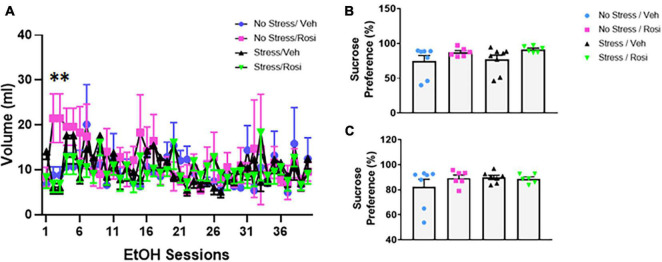
Stress induced ethanol consumption: Beginning PND 52 animals underwent 40 sessions of voluntary 20% ethanol exposure. There was a significant treatment effect driven by session 2 and 3 [ANOVA, *F*(39,858) = 1.939, *p* = 0.0006 **(A)**]. *Post hoc* analysis revealed significant differences between treatment groups during session 2 (No stress + VEH vs. No stress + ROSI, *p* = 0.0479; No stress + ROSI vs. Stress + VEH, *p* = 0.0098; and No stress + ROSI vs. Stress + ROSI, *p* = 0.0222) and session 3 (No stress + VEH vs. No stress + ROSI, *p* = 0.0479; No stress + ROSI vs. Stress + VEH, *p* = 0.0098; No stress + ROSI vs. Stress + ROSI, *p* = 0.0222). Sucrose preference was assessed before the ethanol preference test at PND 51 **(B)** and after the ethanol preference test at PND 149 **(C)**. There were no differences in sucrose preference at either time point when comparing stress group and drug treatment [ANOVA *F*(3,23) = 1.881, *p* = 0.1610 **(B)** and *F*(3,22) = 0.9711, *p* = 0.4241 **(C)**]. *n* = 6–8 per treatment group. Figures show mean ± SEM. ^∗^ = *p* < 0.05.

During the forced swim there was no overall effect on immobility in either the pre-test time immobile or swim time [ANOVA, *F*(3,23) = 2.547, *p* = 0.0809, *p* = 0.0809, Figures 5A,B, respectively], however, there was an overall effect of post-test [ANOVA, *F*(3,23) = 3.307, *p* = 0.0381, Figures 5C,D]. *Post hoc* analysis revealed that animals that underwent no stress but received ROSI (No stress + ROSI) spent significantly less time spent immobile during post-test when compared to the Stress + VEH group (Tukey’s *post hoc*, *p* = 0.0314, Figure 5C) and significantly more time swimming (Tukey’s *post hoc*, *p* = 0.314, Figure 5D). We tested sucrose preference before the ethanol self-administration test at PND 51 and after the ethanol self-administration at PND 149 and found no differences in sucrose consumption when comparing stress group or drug treatment [ANOVA *F*(3,23) = 1.881, *p* = 0.1610, Figure 6B and *F*(3,22) = 0.9711, *p* = 0.4241, Figure 6C, respectively].

### Addiction-Like Behavior: Ethanol Self-Administration

On PND 52 we provided animals access to 20% ethanol in a two-bottle choice paradigm to determine whether unpredictable chronic stress would influence ethanol consumption. Repeated measures ANOVA revealed an overall treatment effect [ANOVA, *F*(39,858) = 1.939, *p* = 0.0006, Figure 6A]. Tukey’s *post hoc* analyses demonstrated that there were significant differences between treatment groups during session 2 (No stress + VEH vs. No stress + ROSI, *p* = 0.0479; No stress + ROSI vs. Stress + VEH, *p* = 0.0098; and No stress + ROSI vs. Stress + ROSI, *p* = 0.0222) and session 3 (No stress + VEH vs. No stress + ROSI, *p* = 0.0479; No stress + ROSI vs. Stress + VEH, *p* = 0.0098; No stress + ROSI vs. Stress + ROSI, *p* = 0.0222). There were no differences in ethanol consumption when comparing No Stress + VEH vs. Stress + VEH (*p* > 0.05) or No Stress + VEH vs. Stress + ROSI (*p* > 0.05), suggesting no overall effect of stress on ethanol consumption.

### Novelty Seeking: Social Context

**FIGURE 7:**
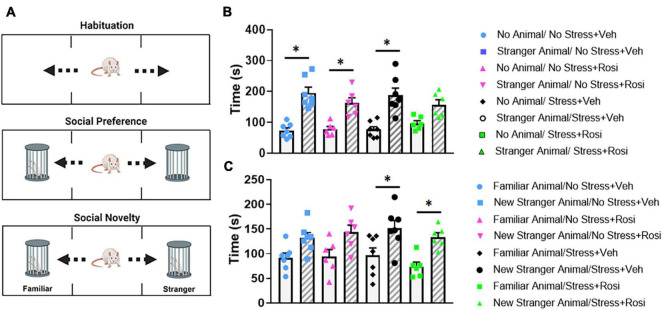
Novelty seeking: Social paradigm. Representative schematic of the phases for the social interaction paradigm **(A)**, there is a habituation phase in which animals get to explore the apparatus. In phase 1, animals had the option of interacting with the empty animal holder or a stranger rat of the same sex. In phase 2, animals had the option to interact with the now familiar rat (from phase 1) or novel rat. There was an overall significant effect of stranger presence [ANOVA, *F*(7,44) = 13.18, *p* < 0.0001 **(B)**]. The No stress + VEH, No stress + ROSI, and Stress + VEH groups all spent significantly more time with the stranger rat when compared to the empty enclosure (*p* < 0.0001, *p* = 0.007, *p* < 0.0001, respectively); however, Stress + ROSI animals did not (*p* = 0.1245). In phase 2, animals had the choice of spending time with the stranger rat (that was now considered familiar) or spending time with the “new” stranger rat **(C)**. There was an overall effect of “new” stranger [ANOVA *F*(7,44) = 5.159, *p* = 0.0002]. The Stress + VEH and Stress + ROSI groups both spent significantly more time with the new stranger rat compared with the familiar rat (*p* = 0.0447, *p* = 0.0450, respectively), while the No stress + VEH and No stress + ROSI did not (*p* = 0.2970, *p* = 0.1707, respectively). *n* = 6–8 per treatment group. Figures show mean ± SEM. ^∗^ = *p* < 0.05.

To determine how stress impacts social interaction with stranger animals, we ran the test animals through a social interaction task (Figure 7A). In this task, test animals initially had the option of interacting with the empty animal holder or a stranger rat of the same sex within an animal holder on the opposite side of the apparatus (phase 1). Within phase 1, there was an overall significant effect of stranger presence [ANOVA, *F*(7,44) = 13.18, *p* < 0.0001, Figure 7B]. *Post hoc* analysis revealed that the No stress + VEH, No stress + ROSI, and Stress + VEH groups all spent significantly more time with the stranger rat when compared to the empty enclosure (Tukey’s *post hoc*, *p* < 0.0001, *p* = 0.007, *p* < 0.0001); however, Stress + ROSI animals did not spend significantly more time with the stranger rat when compared to the empty enclosure (*p* = 0.1245). In the second phase of the experiment, test animals had the choice of spending time with the stranger rat (that was now considered familiar) or spending time with the “new” stranger rat. There was an overall effect of “new” stranger [ANOVA *F*(7,44) = 5.159, *p* = 0.0002, Figure 7C]. *Post hoc* analysis revealed that, while the No stress + VEH and No stress + ROSI groups spent more time with the new stranger rat when compared with the familiar rat, neither measure reached significance (Tukey’s *post hoc p* = 0.2970, *p* = 0.1707, respectively). However, both the Stress + VEH and Stress + ROSI groups spent significantly more time with the new stranger rat compared with the familiar rat (*p* = 0.0447, *p* = 0.0450, respectively), suggesting that the stress induction paradigm increased novelty seeking in the social context that was not attenuated by the co-administration of rosiglitazone.

### Novelty Seeking: Novel Object Recognition

**FIGURE 8:**
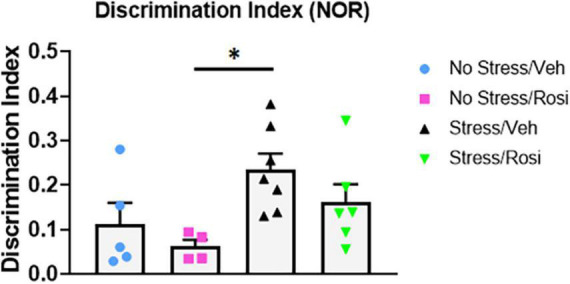
Novelty seeking: Novel object recognition (NOR). Here, we used the NOR to assess working memory and preference for novelty. There was an overall effect of object preference in the discrimination index [ANOVA, *F*(3,18) = 3.459, *p* = 0.0383, Figure 8]. The Stress + VEH group had a higher discrimination index when compared to the No stress + ROSI group (*p* = 0.0361) indicating enhanced working memory and/or a preference for novelty in the Stress + VEH group that was attenuated by the co-administration of rosiglitazone (Stress + VEH vs. Stress + ROSI, *p* = 0.4914 and Stress + ROSI vs. No stress + ROSI, p = *0*.3724). Two animals were excluded from the analysis for moving one or more objects. *n* = 4–7 per treatment group. Figures show mean ± SEM. ^∗^ = *p* < 0.05.

Novel object recognition is a frequently used task for assessing working memory, however, results can be confounded when there are changes in baseline novelty seeking. Here, we used the NOR to assess working memory and preference for novelty. We found no difference in total inspection time (object exploration combined) during the AA or AB trial across stress or treatment groups [ANOVA, *F*(3,18) = 2.371, *p* = 0.1044 (AA trial, data not shown) and ANOVA, *F*(3,18) = 0.6306, *p* = 0.6047 (AB trial, data not shown)]. However, there was an overall effect of object preference in the discrimination index [ANOVA, *F*(3,18) = 3.459, *p* = 0.0383, Figure 8]. *Post hoc* analysis revealed a significant increase in object discrimination index in the Stress + VEH group when compared to the No stress + ROSI group (*p* = 0.0361), indicating enhanced working memory and/or a preference for novelty in the Stress + VEH group that was attenuated by the co-administration of rosiglitazone (Stress + VEH vs. Stress + ROSI, *p* = 0.4914 and Stress + ROSI vs. No stress + ROSI, *p* = 0.3724).

## Discussion

The aims of this study were to investigate the effects of chronic unpredictable stress during adolescence on anxiety, risk taking, depression, ethanol self-administration, and novelty seeking, and to determine whether administration of ROSI can attenuate the stress-induced behavioral changes that emerge. Overall, our findings indicate that animals that undergo stress exhibit confounding effects in two behavioral measures of anxiety and risk taking (as indicated by an increase in the time spent in the open arm of the elevated plus maze and an increase in thigmotaxis in the open field test). Stressed animals demonstrated an increase in novelty seeking in the social context (social interaction) and in the context of object novelty (novel object recognition) with no signs of a depression-like behavioral phenotype (forced swim and sucrose preference). The administration of ROSI resulted in the attenuation of risk-taking behavior in the elevated plus maze and reversal of novelty seeking in the novel object recognition but no effect in the open field and social interaction task, demonstrating task-specific efficacy of this drug in preventing stress-induced behavioral changes.

Social behavior, risk taking, and novelty seeking are classic behavioral characteristics that emerge during adolescence (Spear, 2000). Due to ongoing brain development and maturation during this period of time it has been described as a critical period of vulnerability for addiction and stress (Wills et al., 1994; Arnsten and Shansky, 2004; Crews et al., 2007). The findings in the current study demonstrate that adolescent stress increases risk taking behavior in the EPM, consistent with a number of previous studies (McCormick et al., 2008; Peleg-Raibstein and Feldon, 2011; Toledo and Sandi, 2011; Chaby et al., 2013). Using an adolescent social stress paradigm, McCormick et al. (2008) demonstrated that female rats in estrous show reduced anxiety in the EPM. Additionally, Peleg-Raibstein and Feldon (2011) used a 5 day unpredictable stress paradigm in adolescent and adult male C57BL/6 mice and demonstrated reduced freezing behavior and increased open arm time in the EPM but only when stressed in adolescence. When stressed in adulthood, there was no effect on risk taking behavior (they did not assess females) (Peleg-Raibstein and Feldon, 2011). These data are consistent with previous findings from Toledo and Sandi (2011) in which they used an adolescent rat predator odor stress model (PND 28–42) and showed that adolescent stress increased risk taking behavior in the EPM in both male and female rats. Interestingly, these authors also found indications of mild anxiety in the open field as indicated by stressed animals taking significantly longer to enter the center zone of the apparatus. This is somewhat consistent with our current study in which we find stressed animals show increased risk taking in the EPM but spent more time in the thigmotaxis zone in the open field. It should be noted that if an animal displays risk taking behavior, it does not exclude the presence of anxiety; the presence of anxiety simply influences the risk/reward assessment that the animals perform that will be contingent on the current environmental conditions. These data are further complicated due to a lack of difference between Stress + VEH and Stress + ROSI groups in multiple behavioral measures. However, in the EPM, there is also no significant difference between No stress + VEH and Stress + ROSI. This is due to a partial reduction in open arm time in the Stress + ROSI group compared to Stress + VEH, however, this reduction is insufficient to reach No stress + VEH levels. The open arm time for the Stress + ROSI group resides between the two groups (Stress + VEH and No stress + VEH). These partial effects can also be observed in the open arm distance in the EPM and in the NOR task. Given the presence of this effect over multiple behavioral domains it is possible that this lack of significant effect may be due to the dose of ROSI selected (10 mg/kg, i.g.) since previous studies have used doses ranging from 3 to 20 mg/kg i.g. (Pipatpiboon et al., 2012; Zong et al., 2018; Zhang et al., 2020).

The release of cortisol can occur during a bout of anxiety and can actually increase risk-taking behavior in men (Kluen et al., 2017). The cortisol measures from our current study were obtained immediately following the stress paradigm and show increased cortisol levels in response to adolescent chronic stress. However, administration of ROSI during stress attenuated this effect. ROSI administration also attenuated the stress-induced increased risk taking behavior in the EPM, suggesting that ROSI could be preventing stress-induced circuit remodeling that is occurring through complex neuro-immune-endocrine interactions. These interactions likely involve the upregulation of cortisol and the induction of cytokine release through neuroimmune activation (Haddad et al., 2002; Wiranowska and Plaas, 2008).

The relationship between ROSI and neuroendocrine suppression is not new. ROSI has been shown to downregulate HPA axis hyperactivity in diabetic rats *via* a mechanism dependent on PI3K activation in pituitary and adrenal glands (Torres et al., 2016). ROSI has also been used in diabetic rats to treat adrenal hypertrophy and hypercorticoidism (Ventura et al., 2020), while Goodson et al. (2017) used a chronic variable stress model to demonstrate that administration of ROSI blunted the stress-induced increase in circulating basal cortisol. Here we suggest that blocking stress-induced HPA hyperactivity suppresses downstream neuroimmune modulation and the emergence of maladaptive behavior. The actions by which rosiglitazone mediates its anti-inflammatory effects are complicated because PPARγ are expressed by glial cells and neurons, suggesting complex cell-cell neuroimmune interactions may be involved in rosiglitazone’s mechanisms of action. Recent findings directly support a role for microglia in the actions of rosiglitazone. Carta et al. (2011) demonstrated that rosiglitazone was able to modulate PPARγ receptor expression specifically in microglia without impacting neuronal receptor levels to attenuate neuroinflammation and degeneration in a model of Parkinson’s disease. Further work suggests that the anti-inflammatory action may be through suppression of TNFα (Carta et al., 2011; Liu et al., 2016) or possibly due to rosiglitazone’s ability to drive a microglial switch from a neuroinflammatory activation state to a neuroprotective activation state (Subramaniam and Federoff, 2017; Ji et al., 2018). It may be interesting in future studies to assess the relationship between cortisol response and immune-neuronal-remodeling in the context of risky decision making to elucidate whether blunted cortisol response during the stressful task contributes to the increase in risk taking behavior observed following adolescent stress and whether ROSI administration during or after stress induction can attenuate such an effect.

While we consider the EPM to be a measure of risk taking, we must also consider the contributions of impulsivity and novelty in the context of exploration, either of which could contribute to changes in EPM and open field exploration. Although impulsivity was not assessed in this study, we did assess two different kinds of novelty seeking. We found that adolescent rats exposed to stress during adolescence demonstrate increased novelty seeking in both the social interaction task (social novelty) and the NOR task (object novelty). While the NOR task is typically used as a measure of working memory, it is also a good indicator of novelty preference. In the NOR task used for this study, the stress group spent significantly more time with the novel object than the familiar object compared to the no stress group that received ROSI. This is consistent with Toledo and Sandi (2011), who demonstrated similar increases in stress-dependent novelty seeking using a novel object test. However, novelty seeking was not limited to objects in the current study. When faced with the option to spend time with a familiar rat or a stranger rat in the social interaction task, the stress animals spent significantly more time with the stranger, demonstrating a preference for social novelty. These data become even more interesting when we consider the effect of ROSI administration on novelty seeking in both paradigms. While in the context of social novelty there was no effect of ROSI on preference for the stranger animal, in the NOR there was a significant reduction in discrimination index when ROSI was administered, as demonstrated by a decrease in time spent with the novel object. As previously discussed, disruption of mPFC function can not only decrease anxiety-like behavior but can also increase social interaction time (Gonzalez et al., 2000; Lacroix et al., 2000; Shah and Treit, 2003). This occurs through the modulation of cortisol, suggesting once again a role for HPA axis and cortisol dysregulation following adolescent stress that can be attenuated (in part) by administration of ROSI. These data suggest that the social reward/novelty pathway may be more resistant to adolescent stress-induced remodeling. Further work is required to understand the nuances of novelty seeking in the context of social reward versus object exploration and how ROSI may selectively attenuate one but not the other. However, recent work suggests that unique pathways inside and outside the classic mesolimbic pathway may drive social reward (Huang et al., 2020; Hu et al., 2021) and likely involve mPFC modulation of dopamine regulation (Watson et al., 2012).

Despite adolescent stress exacerbating risk taking and novelty seeking, this did not manifest in the context of increased EtOH consumption. However, ROSI alone (no stress animals) resulted in an increased in EtOH consumption across day 2 and 3 of self-administration which then normalized to the level of all other treatment and stress groups by day 4. Many times when animals self-administer more drug early in a paradigm it is associated with novelty seeking and/or impulsivity. This has typically been the case when comparing adolescent and adult rodents, with adolescent rodents typically demonstrating more consumption or nose poking (in reward tasks) than their older counterparts (Spear, 2000; Romer, 2010; Doremus-Fitzwater et al., 2012). However, ROSI animals do not demonstrate more novelty seeking or risk taking in the EPM and OF. Moreover, if this was simply a response to taste novelty we would expect to see similar differences on the single day sucrose exposure, which we do not, thus making it unlikely that ROSI is inducing increased novelty seeking or risk taking resulting in early increases in EtOH consumption. Regardless of the reasons for increased EtOH administration during day 2 and 3, it did not persist throughout the remaining sessions.

The lack of stress effect on alcohol consumption was surprising since risk taking and novelty seeking are strongly associated with alcohol and substance abuse use and escalation in both humans and animal models (Wingo et al., 2016). It is possible that, despite using a chronic stress paradigm, the mildness of the stressors in the current study produces less robust behavioral and neuroendocrine effects that would be sufficient to manifest as increased alcohol consumption. Regardless, we cannot rule out that animals undergoing this adolescent stress paradigm may have increased abuse liability toward other drugs of abuse or gambling tasks, but clearly there is no generalized reward sensitivity as indicated by no differences in sucrose or alcohol consumption following adolescent stress. However, previous studies have shown that chronic stress during adolescence enhances locomotor sensitization to amphetamine (Mathews et al., 2008) and cocaine (Lepsch et al., 2005) in both male and female rats.

In conclusion, these findings support the growing data demonstrating the vulnerability of the adolescent brain to stressful experiences. These data also show a potential role for the PPARγ pathway in novelty seeking and risk taking behaviors that may be related to neuro-immune-endocrine processes. Further work will be necessary to delineate the role of these pathways and determine the specific downstream cellular and molecular changes that may be involved.

## Data Availability Statement

The original contributions presented in the study are included in the article/supplementary material, further inquiries can be directed to the corresponding author.

## Ethics Statement

The animal study was reviewed and approved by Marshall University Institutional Animal Care and Use Committee.

## Author Contributions

M-LR designed the studies and wrote the manuscript. All authors collected and analyzed the data, and edited and approved the manuscript. HS and M-LR interpreted the data.

## Author Disclaimer

The content is solely the responsibility of the authors and does not necessarily represent the official views of theNational Institutes of Health or the Department of Veterans Affairs.

## Conflict of Interest

The authors declare that the research was conducted in the absence of any commercial or financial relationships that could be construed as a potential conflict of interest.

## Publisher’s Note

All claims expressed in this article are solely those of the authors and do not necessarily represent those of their affiliated organizations, or those of the publisher, the editors and the reviewers. Any product that may be evaluated in this article, or claim that may be made by its manufacturer, is not guaranteed or endorsed by the publisher.

## References

[B1] al’Absi M. Ginty A. T. Lovallo W. R. (2021). Neurobiological mechanisms of early life adversity, blunted stress reactivity and risk for addiction. *Neuropharmacology* 188:108519. 10.1016/j.neuropharm.2021.108519 33711348 PMC9195251

[B2] Alloy L. B. Abramson L. Y. Walshaw P. D. Keyser J. Gerstein R. K. (2006). A cognitive vulnerability-stress perspective on bipolar spectrum disorders in a normative adolescent brain, cognitive, and emotional development context. *Dev. Psychopathol.* 18 1055–1103. 10.1017/S0954579406060524 17064429

[B3] Arnsten A. F. Shansky R. M. (2004). Adolescence: vulnerable period for stress-induced prefrontal cortical function? Introduction to part IV. *Ann. N. Y. Acad. Sci.* 1021 143–147. 10.1196/annals.1308.017 15251883

[B4] Bernardo A. Minghetti L. (2008). Regulation of glial cell functions by PPAR-gamma natural and synthetic agonists. *PPAR Res.* 2008:864140. 10.1155/2008/864140 18464925 PMC2367430

[B5] Bouhlel M. A. Derudas B. Rigamonti E. Dièvart R. Brozek J. Haulon S. (2007). PPARgamma activation primes human monocytes into alternative M2 macrophages with anti-inflammatory properties. *Cell Metab.* 6 137–143. 10.1016/j.cmet.2007.06.010 17681149

[B6] Carpenter L. L. Gawuga C. E. Tyrka A. R. Lee J. K. Anderson G. M. Price L. H. (2010). Association between plasma IL-6 response to acute stress and early-life adversity in healthy adults. *Neuropsychopharmacology* 35 2617–2623. 10.1038/npp.2010.159 20881945 PMC2978751

[B7] Carroll D. Ginty A. T. Whittaker A. C. Lovallo W. R. de Rooij S. R. (2017). The behavioural, cognitive, and neural corollaries of blunted cardiovascular and cortisol reactions to acute psychological stress. *Neurosci. Biobehav. Rev.* 77 74–86. 10.1016/j.neubiorev.2017.02.025 28254428 PMC6741350

[B8] Carta A. R. Frau L. Pisanu A. Wardas J. Spiga S. Carboni E. (2011). Rosiglitazone decreases peroxisome proliferator receptor-γ levels in microglia and inhibits TNF-α production: new evidences on neuroprotection in a progressive Parkinson’s disease model. *Neuroscience* 194 250–261. 10.1016/j.neuroscience.2011.07.046 21839812

[B9] Chaby L. E. Cavigelli S. A. White A. Wang K. Braithwaite V. A. (2013). Long-term changes in cognitive bias and coping response as a result of chronic unpredictable stress during adolescence. *Front. Hum. Neurosci.* 7:328. 10.3389/fnhum.2013.00328 23847501 PMC3701140

[B10] Chinetti G. Fruchart J. C. Staels B. (2000). Peroxisome proliferator-activated receptors (PPARs): nuclear receptors at the crossroads between lipid metabolism and inflammation. *Inflamm. Res.* 49 497–505. 10.1007/s000110050622 11089900

[B11] Chinetti G. Fruchart J. C. Staels B. (2003). Peroxisome proliferator-activated receptors: new targets for the pharmacological modulation of macrophage gene expression and function. *Curr. Opin. Lipidol.* 14 459–468. 10.1097/00041433-200310000-00006 14501584

[B12] Crews F. He J. Hodge C. (2007). Adolescent cortical development: a critical period of vulnerability for addiction. *Pharmacol. Biochem. Behav.* 86 189–199. 10.1016/j.pbb.2006.12.001 17222895 PMC11646682

[B13] Cullingford T. E. Bhakoo K. Peuchen S. Dolphin C. T. Patel R. Clark J. B. (1998). Distribution of mRNAs encoding the peroxisome proliferator-activated receptor alpha, beta, and gamma and the retinoid X receptor alpha, beta, and gamma in rat central nervous system. *J. Neurochem.* 70 1366–1375. 10.1046/j.1471-4159.1998.70041366.x 9523552

[B14] Doremus-Fitzwater T. L. Barreto M. Spear L. P. (2012). Age-related differences in impulsivity among adolescent and adult Sprague-Dawley rats. *Behav. Neurosci.* 126 735–741. 10.1037/a0029697 22889309 PMC3583377

[B15] Duffy K. A. McLaughlin K. A. Green P. A. (2018). Early life adversity and health-risk behaviors: proposed psychological and neural mechanisms. *Ann. N. Y. Acad. Sci.* 1428 151–169. 10.1111/nyas.13928 30011075 PMC6158062

[B16] Eissa Ahmed A. A. Al-Rasheed N. M. Al-Rasheed N. M. (2009). Antidepressant-like effects of rosiglitazone, a PPARγ agonist, in the rat forced swim and mouse tail suspension tests. *Behav. Pharmacol.* 20 635–642. 10.1097/FBP.0b013e328331b9bf 19745723

[B17] Engel M. L. Gunnar M. R. (2020). “Chapter Three - The development of stress reactivity and regulation during human development,” in *International Review of Neurobiology*, eds Clow A. Smyth N. (Cambridge: Academic Press), 41–76. 10.1016/bs.irn.2019.11.003 32204834

[B18] Esquivel-Rendón E. Vargas-Mireles J. Cuevas-Olguín R. Miranda-Morales M. Acosta-Mares P. García-Oscos F. (2019). Interleukin 6 dependent synaptic plasticity in a social defeat-susceptible prefrontal cortex circuit. *Neuroscience* 414 280–296. 10.1016/j.neuroscience.2019.07.002 31301368

[B19] Fernandes J. Gupta G. L. (2019). N-acetylcysteine attenuates neuroinflammation associated depressive behavior induced by chronic unpredictable mild stress in rat. *Behav. Brain Res.* 364 356–365. 10.1016/j.bbr.2019.02.025 30772427

[B20] Fu R. Gregor D. Peng Z. Li J. Bekker A. Ye J. (2015). Chronic intermittent voluntary alcohol drinking induces hyperalgesia in Sprague-Dawley rats. *Int. J. Physiol. Pathophysiol. Pharmacol.* 7 136–144. 26823962 PMC4697669

[B21] Gonzalez L. E. Rujano M. Tucci S. Paredes D. Silva E. Alba G. (2000). Medial prefrontal transection enhances social interaction. I: behavioral studies. *Brain Res.* 887 7–15. 10.1016/s0006-8993(00)02931-0 11134584

[B22] Goodson M. L. Packard A. E. B. Buesing D. R. Maney M. Myers B. Fang Y. (2017). Chronic stress and Rosiglitazone increase indices of vascular stiffness in male rats. *Physiol. Behav.* 172 16–23. 10.1016/j.physbeh.2016.03.031 27040922 PMC5045780

[B23] Guo M. Li C. Lei Y. Xu S. Zhao D. Lu X. Y. (2017). Role of the adipose PPARγ-adiponectin axis in susceptibility to stress and depression/anxiety-related behaviors. *Mol. Psychiatry* 22 1056–1068. 10.1038/mp.2016.225 27956741 PMC5468488

[B24] Haddad J. J. Saadé N. E. Safieh-Garabedian B. (2002). Cytokines and neuro-immune-endocrine interactions: a role for the hypothalamic-pituitary-adrenal revolving axis. *J. Neuroimmunol.* 133 1–19. 10.1016/s0165-5728(02)00357-0 12446003

[B25] Herbison C. E. Allen K. Robinson M. Newnham J. Pennell C. (2017). The impact of life stress on adult depression and anxiety is dependent on gender and timing of exposure. *Dev. Psychopathol.* 29 1443–1454. 10.1017/S0954579417000372 28397629

[B26] Horak N. S. Eagle G. Stein D. J. Lochner C. (2021). Gambling disorder and childhood trauma: a complex association. *J. Gambl. Stud.* 37 515–528. 10.1007/s10899-020-09983-w 33006105

[B27] Hu R. K. Zuo Y. Ly T. Wang J. Meera P. Wu Y. E. (2021). An amygdala-to-hypothalamus circuit for social reward. *Nat. Neurosci.* 24 831–842. 10.1038/s41593-021-00828-2 33820999 PMC8236486

[B28] Huang W. C. Zucca A. Levy J. Page D. T. (2020). Social behavior is modulated by valence-encoding mPFC-Amygdala Sub-circuitry. *Cell Rep.* 32:107899. 10.1016/j.celrep.2020.107899 32668253 PMC7410267

[B29] Ji J. Xue T. F. Guo X. D. Yang J. Guo R. B. Wang J. (2018). Antagonizing peroxisome proliferator-activated receptor γ facilitates M1-to-M2 shift of microglia by enhancing autophagy via the LKB1-AMPK signaling pathway. *Aging Cell* 17:e12774. 10.1111/acel.12774 29740932 PMC6052482

[B30] Kaidanovich-Beilin O. Lipina T. Vukobradovic I. Roder J. Woodgett J. R. (2011). Assessment of social interaction behaviors. *J. Vis. Exp.* 48:2473. 10.3791/2473 21403628 PMC3197404

[B31] Kluen L. M. Agorastos A. Wiedemann K. Schwabe L. (2017). Cortisol boosts risky decision-making behavior in men but not in women. *Psychoneuroendocrinology* 84 181–189. 10.1016/j.psyneuen.2017.07.240 28750292

[B32] Lacroix L. Spinelli S. Heidbreder C. A. Feldon J. (2000). Differential role of the medial and lateral prefrontal cortices in fear and anxiety. *Behav. Neurosci.* 114 1119–1130. 10.1037//0735-7044.114.6.1119 11142644

[B33] LeMoult J. Humphreys K. L. Tracy A. Hoffmeister J. A. Ip E. Gotlib I. H. (2020). Meta-analysis: exposure to early life stress and risk for depression in childhood and adolescence. *J. Am. Acad. Child Adolesc. Psychiatry* 59 842–855. 10.1016/j.jaac.2019.10.011 31676392 PMC11826385

[B34] Lepsch L. B. Gonzalo L. A. Magro F. J. Delucia R. Scavone C. Planeta C. S. (2005). Exposure to chronic stress increases the locomotor response to cocaine and the basal levels of corticosterone in adolescent rats. *Addict. Biol.* 10 251–256. 10.1080/13556210500269366 16109586

[B35] Liu H. Rose M. E. Culver S. Ma X. Dixon C. E. Graham S. H. (2016). Rosiglitazone attenuates inflammation and CA3 neuronal loss following traumatic brain injury in rats. *Biochem. Biophys. Res. Commun.* 472 648–655. 10.1016/j.bbrc.2016.03.003 26947332

[B36] López-López A. L. Jaime H. B. Escobar Villanueva M. D. C. Padilla M. B. Palacios G. V. Aguilar F. J. A. (2016). Chronic unpredictable mild stress generates oxidative stress and systemic inflammation in rats. *Physiol. Behav.* 161 15–23. 10.1016/j.physbeh.2016.03.017 27063246

[B37] Mathews I. Z. Mills R. G. McCormick C. M. (2008). Chronic social stress in adolescence influenced both amphetamine conditioned place preference and locomotor sensitization. *Dev. Psychobiol.* 50 451–459. 10.1002/dev.20299 18551462

[B38] McCormick C. M. Smith C. Mathews I. Z. (2008). Effects of chronic social stress in adolescence on anxiety and neuroendocrine response to mild stress in male and female rats. *Behav. Brain Res.* 187 228–238. 10.1016/j.bbr.2007.09.005 17945360

[B39] Mograbi K. M. Suchecki D. da Silva S. G. Covolan L. Hamani C. (2020). Chronic unpredictable restraint stress increases hippocampal pro-inflammatory cytokines and decreases motivated behavior in rats. *Stress* 23 427–436. 10.1080/10253890.2020.1712355 31928117

[B40] Moreno S. Farioli-Vecchioli S. Cerù M. P. (2004). Immunolocalization of peroxisome proliferator-activated receptors and retinoid X receptors in the adult rat CNS. *Neuroscience* 123 131–145. 10.1016/j.neuroscience.2003.08.064 14667448

[B41] Munjiza A. Kostic M. Pesic D. Gajic M. Markovic I. Tosevski D. L. (2018). Higher concentration of interleukin 6 - A possible link between major depressive disorder and childhood abuse. *Psychiatry Res.* 264 26–30. 10.1016/j.psychres.2018.03.072 29626828

[B42] Peleg-Raibstein D. Feldon J. (2011). Differential effects of post-weaning juvenile stress on the behaviour of C57BL/6 mice in adolescence and adulthood. *Psychopharmacology* 214 339–351. 10.1007/s00213-010-1991-8 20803000

[B43] Pipatpiboon N. Pratchayasakul W. Chattipakorn N. Chattipakorn S. C. (2012). PPARγ agonist improves neuronal insulin receptor function in hippocampus and brain mitochondria function in rats with insulin resistance induced by long term high-fat diets. *Endocrinology* 153 329–338. 10.1210/en.2011-1502 22109891

[B44] Richter B. Bandeira-Echtler E. Bergerhoff K. Clar C. Ebrahim S. H. (2007). Rosiglitazone for type 2 diabetes mellitus. *Cochrane Database Syst. Rev.* 2007:CD006063. 10.1002/14651858.CD006063.pub2 PMC7389529 17636824

[B45] Romer D. (2010). Adolescent risk taking, impulsivity, and brain development: implications for prevention. *Dev. Psychobiol.* 52 263–276. 10.1002/dev.20442 20175097 PMC3445337

[B46] Sequeira-Cordero A. Salas-Bastos A. Fornaguera J. Brenes J. C. (2019). Behavioural characterisation of chronic unpredictable stress based on ethologically relevant paradigms in rats. *Sci. Rep.* 9:17403. 10.1038/s41598-019-53624-1 31758000 PMC6874551

[B47] Shah A. A. Treit D. (2003). Excitotoxic lesions of the medial prefrontal cortex attenuate fear responses in the elevated-plus maze, social interaction and shock probe burying tests. *Brain Res.* 969 183–194. 10.1016/s0006-8993(03)02299-6 12676379

[B48] Simms J. A. Steensland P. Medina B. Abernathy K. E. Chandler L. J. Wise R. (2008). Intermittent access to 20% ethanol induces high ethanol consumption in Long-Evans and Wistar rats. *Alcohol. Clin. Exp. Res.* 32 1816–1823. 10.1111/j.1530-0277.2008.00753.x 18671810 PMC3151464

[B49] Sinha R. (2008). Chronic stress, drug use, and vulnerability to addiction. *Ann. N. Y. Acad. Sci.* 1141 105–130. 10.1196/annals.1441.030 18991954 PMC2732004

[B50] Spear L. (2000). The adolescent brain and age-related behavioral manifestations. *Neurosci. Biobehav. Rev.* 24 417–463. 10.1016/s0149-7634(00)00014-2 10817843

[B51] Standiford T. J. Keshamouni V. G. Reddy R. C. (2005). Peroxisome proliferator-activated receptor-{gamma} as a regulator of lung inflammation and repair. *Proc. Am. Thorac. Soc.* 2 226–231. 10.1513/pats.200501-010ac 16222042

[B52] Subramaniam S. R. Federoff H. J. (2017). Targeting microglial activation states as a therapeutic avenue in Parkinson’s disease. *Front. Aging Neurosci.* 9:176. 10.3389/fnagi.2017.00176 28642697 PMC5463358

[B53] Swartzwelder N. A. Risher M. L. Abdelwahab S. H. D’Abo A. Rezvani A. H. Levin E. D. (2012). Effects of ethanol, Δ^9^-tetrahydrocannabinol, or their combination on object recognition memory and object preference in adolescent and adult male rats. *Neurosci. Lett.* 527 11–15. 10.1016/j.neulet.2012.08.037 22959891 PMC3477605

[B54] Teixeira C. A. B. Lasiuk G. Barton S. Fernandes M. N. F. Gherardi-Donato E. (2017). An exploration of addiction in adults experiencing early-life stress: a metasynthesis. *Rev. Lat. Am. Enfermagem* 25:e2939. 10.1590/1518-8345.2026.2939 29020127 PMC5635699

[B55] Toledo M. Sandi C. (2011). Stress during adolescence increases novelty seeking and risk-taking behavior in male and female rats. *Front. Behav. Neurosci.* 5:17. 10.3389/fnbeh.2011.00017 21519389 PMC3078747

[B56] Torres R. C. Magalhães N. S. Pm E. S. Martins M. A. Carvalho V. F. (2016). Activation of PPAR-γ reduces HPA axis activity in diabetic rats by up-regulating PI3K expression. *Exp. Mol. Pathol.* 101 290–301. 10.1016/j.yexmp.2016.10.002 27725163

[B57] Ventura R. D. Chaves A. S. Magalhães N. S. Gonzalez F. B. Pacini M. F. Pérez A. R. (2020). Activation of PPARγ reduces N-acetyl-cysteine -induced hypercorticoidism by down-regulating MC2R expression into adrenal glands. *Free Radic. Biol. Med.* 156 137–143. 10.1016/j.freeradbiomed.2020.06.008 32574682

[B58] Wagstaff A. J. Goa K. L. (2002). Rosiglitazone: a review of its use in the management of type 2 diabetes mellitus. *Drugs* 62 1805–1837. 10.2165/00003495-200262120-00007 12149047

[B59] Watson D. J. Loiseau F. Ingallinesi M. Millan M. J. Marsden C. A. Fone K. C. (2012). Selective blockade of dopamine D3 receptors enhances while D2 receptor antagonism impairs social novelty discrimination and novel object recognition in rats: a key role for the prefrontal cortex. *Neuropsychopharmacology* 37 770–786. 10.1038/npp.2011.254 22030711 PMC3261029

[B60] Wills T. A. Vaccaro D. McNamara G. (1994). Novelty seeking, risk taking, and related constructs as predictors of adolescent substance use: an application of Cloninger’s theory. *J. Subst. Abuse* 6 1–20. 10.1016/s0899-3289(94)90039-6 8081104

[B61] Wingo T. Nesil T. Choi J. S. Li M. D. (2016). Novelty seeking and drug addiction in humans and animals: from behavior to molecules. *J. Neuroimmune Pharmacol.* 11 456–470. 10.1007/s11481-015-9636-7 26481371 PMC4837094

[B62] Wiranowska M. Plaas A. (2008). Cytokines and extracellular matrix remodeling in the central nervous system. *NeuroImmune Biol.* 6 167–197. 10.1016/s1567-7443(07)10009-0

[B63] Wohleb E. S. Terwilliger R. Duman C. H. Duman R. S. (2018). Stress-induced neuronal colony stimulating factor 1 provokes microglia-mediated neuronal remodeling and depressive-like behavior. *Biol. Psychiatry* 83 38–49. 10.1016/j.biopsych.2017.05.026 28697890 PMC6506225

[B64] Zhang S. M. Cai X. F. Ma Y. L. Lu Q. (2020). Effect of rosiglitazone on myocardial injury in septic rats through NF-κB pathway. *Eur. Rev. Med. Pharmacol. Sci.* 24 452–460. 10.26355/eurrev_202001_19945 31957860

[B65] Zong J. Liao X. Ren B. Wang Z. (2018). The antidepressant effects of rosiglitazone on rats with depression induced by neuropathic pain. *Life Sci.* 203 315–322. 10.1016/j.lfs.2018.04.057 29730170

